# Supplementary material for: Children’s exploratory play tracks the discriminability of hypotheses
Source: Nat Commun. 2021 Jun 14;12:3598. doi: 10.1038/s41467-021-23431-2 (PMC8203670; doi:10.1038/s41467-021-23431-2)
Supplement: Supplementary file 1 — Supplementary Information [file 41467_2021_23431_MOESM1_ESM.docx]

Supplementary Materials for

Children's exploratory play tracks the discriminability of hypotheses.

Siegel, M.H.^†^, Magid, R.^†^, Pelz, M., Tenenbaum, J.B., & Schulz, L.E.

†Both authors contributed equally

Correspondence to: maxs@mit.edu

**This PDF file includes:**

Materials and Methods

Supplementary Text

Table S1-S2

Figure S1

Supplementary Information References

**Supplementary Materials:**

**Preliminary Experiments**

Participants

Sixty children (mean age: 4;6; range: 2;7-6;3) were recruited at a local children’s museum. Fifteen children participated in each study (Object Identity: mean: 4;4, range: 3;0-6;3; Object Number: mean: 3;11, range: 2;7-5;9; Volume Control: mean: 4;11, range: 2;9-6;1; Diverse Actions: mean: 4;10, range: 3;5-5;11).

The same population (drawn from an urban children’s museum) was sampled for all studies reported in this manuscript. While most of the children were white and middle class, a range of ethnicities and socioeconomic backgrounds reflecting the diversity of the local population (47% European American, 24% African American, 9% Asian, 17% Latino, 4% two or more races) and the museum population (29% of museum attendees receive free or discounted admission) were represented. All experiments were approved by an institutional review board for human subjects and all ethical guidelines were followed. The child’s parent or legal guardian was provided with a verbal description of the study. The experimenter answered any questions the parent had. The parent or legal guardian then provided written informed consent allowing the child to participate in the study and be videotaped, consistent with the MIT IRB approval for the study. Children over age seven also provided verbal assent.

Materials

In all studies, two cardboard shoeboxes covered with black electrical tape were used and a large cardboard screen (80 x 60 cm) was used as an occluder. In the *Object Identity* study, a square beanbag and a plastic ball of equal weight were used (5 cm diameter). For the remaining studies, ten colored marbles and two translucent cylindrical tubes were used. Although the children thought the marbles were being poured from the cylinders, they were in fact sealed and the boxes were pre-loaded with two and eight marbles. A stuffed animal bunny was used as a character in the script. In the *Volume Control* experiment, a felt cloth fitted to the bottom of the shoebox was also used.

Procedure

All children were tested individually in a private testing room off of the museum floor. The child and the experimenter sat on opposite sides of a child-sized table. All sessions were videotaped. Children’s responses were coded live by the experimenter and recoded by a coder blind to condition from video using the VCode (*1*) video annotation software.

*Object Identity*

The experimenter placed the pair of boxes on top of the table. The experimenter introduced the beanbag and the plastic ball one at a time (order counterbalanced). She let the child hold each object and commented on their properties as follows: “Look, the beanbag is soft” and “Look, the plastic ball is hard”. To incentivize the child to attend to each object individually and choose one object, she asked the child which of the two objects was his favorite. The experimenter then explained the task: “I'm going to put each one of these things in a different box, and then shake each box! Then we'll listen and try to figure out which box has your favorite thing in it. Do you want to help me figure out which box has your favorite thing in it?” She set up the occluding screen so the child could not see her actions and silently placed each object in one of the two boxes (left/right counterbalanced). The experimenter then removed the screen and said “Okay, one of these two boxes has your favorite thing in it. I’m going to shake the boxes and you try to guess which object has your favorite thing in it.” The experimenter picked up one box and shook it five times. Then she picked up the other box and shook it five times (order counterbalanced). The experimenter then asked, “Which box has your favorite thing in it?”

*Object Number*

The experimenter placed the pair of boxes on top of the table. The experimenter introduced the two cylinders, one of which had two marbles inside and the other of which had eight marbles inside (order counterbalanced). She asked the child to count the number of marbles in each cylinder. Then she introduced the bunny rabbit. The bunny rabbit expressed a preference for either the container with the two marbles or the container with the eight marbles (counterbalanced) saying, “I like this one! This one is my favorite!”

The experimenter then explained the task: “I'm going to pour the two marbles into one of these boxes, and the eight marbles into the other box and then I’m going to shake each box! Do you want to help me figure out which box has Bunny’s favorite marbles inside it?” She set up the occluding screen so the child could not see her actions and made identical sounds by tilting one of the cylinders upside down. (To avoid acoustic cues from her actions, the cylinders were actually sealed and the boxes were pre-loaded with the marbles: left/right and color counterbalanced). The experimenter then removed the screen and said, “Okay, do you remember if Bunny liked the two marbles or the eight marbles better?” All children answered this question correctly. Then the experimenter said, “That’s right! One of these two boxes has two marbles in it and the other one has the eight marbles in it. I’m going to shake the box and you can help me figure out which box Bunny should open.” She shook each box five times (order counterbalanced) and then asked, “Which box does Bunny want to open?”

*Volume Control*

To assess the flexibility of children’s perceptual judgments, and children’s ability to succeed on more complex perceptual identification tasks (closer to the complexity required to assess the information search question of primary interest) we removed differential volume as a cue by adding a felt blanket to the box with more marbles, and tested children a year older. The study was identical to the one described above, except that we inserted a felt cloth into one of the two boxes. After shaking each box five times, children were told, “One of these two boxes has a felt blanket inside along with the marbles. Can you tell me which box has the felt blanket inside?” Children were then reminded that one of the boxes had two marbles inside and one had eight marbles inside and were asked, “Which box does Bunny want to open?”

*Diverse Actions*

All of the previous studies used the same physical manipulation, shaking the box, for all contrasts. It is possible that this simplified the children's task, by allowing children to focus on a single dimension of the sound (e.g., the number of collisions). To address this, we repeated the protocol used in the *Object Number* experiment, but shook the box with two marbles (as before) and gently rocked the box with eight marbles. These diverse actions produced sounds that differed along many dimensions. Gentle rocking and vigorous shaking produce very different sounds even with equal numbers of marbles in the box, thus if children succeed, the perception of numerosity from sound must be relatively robust.

Results

Children performed at ceiling in both the *Object Identity* and *Object Number* experiment: 100% of the children correctly identified the object with their (or the bunny’s) preferred objects. Children performed above chance in both the *Volume Control* (86.7% answered correctly; 95% CI [0.67-1]) and the *Diverse Actions* task (86.7% answered correctly; 95% CI [0.67-1]).

**Experiments 1-3**

*Experiment 1*

Participants

Twenty-four children were recruited from a local children’s museum; eight were excluded from further analysis for preferring the distractor object (see below), resulting in a sample of sixteen children (mean age: 4;7, range: 3;1-6;2). Although we included two-year-olds in the preliminary experiments, we did not include them in the following studies because pilot work established that the task demands (requiring them to represent that one of two items could be placed in each box) were too high.

Materials

The materials used in the preliminary *Object Identity* and *Object Number* experiments were used here for warm-up tasks. (These materials differed in both appearance and acoustic properties from those used in Experiment 1). In Experiment 1, two pencils with a shiny, holographic coating were used as target objects. A standard yellow pencil and a small, cotton-filled fabric cushion were used as distractor objects. A stuffed animal bunny was used to occupy the children’s hands so that they did not reach for the stimuli or interfere with demonstrations.

Procedure

All children were tested individually in a private testing room in the children’s museum. The child and the experimenter sat on opposite sides of a child-sized table. All sessions were videotaped.

The experimenter placed the pair of boxes on top of the table. After the warm-up tasks, children were introduced to two pairs of objects, each of which consisted of a target and a distractor stimulus. The target stimulus (the holographic pencil) was identical across both pairs, and was intended to be more desirable than either distractor. The distractor in the Ambiguous pair was chosen to sound very similar to the target when shaken inside a box (the standard #2 pencil). The distractor in the Unambiguous pair was chosen to sound very different from the target (the cotton pillow).

After introducing the objects in each pair, the experimenter asked the child what her favorite object was in each pair. We required that children preferred the target object in both pairs because the experimental task involved finding an object potentially present in both boxes; additionally, children who preferred a distractor object might simply choose the box it could be in rather than consider both boxes. Children who did not (i.e. preferred one or both of the distractor objects) were excluded and replaced. Eight children were excluded for this reason (three preferred the #2 pencil and five preferred the cotton pillow).

After children picked their favorite objects, the experimenter said, “I’m going to take just one object -- either the shiny pencil or the plain pencil -- and put it in this box here. And then I’m going to take just one object -- either the shiny pencil or the cotton pillow -- and put it in this box here.” The experimenter placed the boxes and objects behind an occluder and silently hid the shiny pencil in each box (left/right and color of boxes counterbalanced). After the objects were hidden, the experimenter removed the occluder and told the child, “Remember, inside this box, there could be either a cool shiny pencil or the pillow” or “Remember inside this box, there could be either a cool shiny pencil or the plain yellow pencil.” (counterbalanced). The experimenter then said, “I’m going to shake each box and then you can choose which box you want to open. You get to take whatever is inside the box home with you.” The experimenter shook each box twice. The experimenter repeated the about the possible contents of each box and then shook each box twice again. She said, “Go ahead, you can choose one of these boxes to open and you get to take home what you find inside.” See Figure 1, main text.

Results

Thirteen out of sixteen children successfully chose the box where the unheard alternative, the pillow, would have been easier to discriminate from the target (81.2%; 95% CI [0.63-1]); the remaining three picked the box where the unheard alternative, the pencil, would have been difficult.

*Experiment 2*

Participants

Based on the results of the preliminary experiments, we estimated the effect size for a single experiment as *f=*0.29. We used the power calculation program G*Power to calculate the planned sample size of for this experiment using *f*=0.29, ɑ = 0.05, and power = 0.80. The projected sample size using these values is 24 participants, which was used for Experiments 2 and 3.

Fifty-two children were recruited; four participants were excluded from analysis, three because of experimenter error and one for inability to understand and follow directions. Twenty-four children were assigned to the *Discrimination* task (mean age: 4;2; range: 3;0-5;4) and twenty-four were assigned to a *Similarity Judgment* task (mean age: 4;8; range: 3;0-6;1).

Materials

The materials used in the *Object Identity* experiment were used for a warm-up task. Additionally, in Experiment 2, one large (approximately 8 cm by 5 cm) and six small (approximately 3 cm by 2 cm) plastic elephants were used. A small plastic pig (approximately 3 cm by 2 cm) was also used. A transparent, hexagonally partitioned container was used as the baby elephants’ home. A stuffed animal bunny was used to occupy children’s hands so that they did not reach for the stimuli or interfere with the demonstrations.

Procedure

All children were tested individually in a private testing room off of the museum floor. The child and the experimenter sat on opposite sides of a child-sized table. All sessions were videotaped.

The *Object Identity* task from the preliminary studies (see SI) was used as a warm-up task. The *Discrimination* task was identical to Experiment 1 except as follows. The experimenter showed participants a clear plastic container partitioned into six compartments, five of which contained small plastic elephants. The experimenter described the container as an elephant house, and said that one of the baby elephants had gone missing and asked participants to help find the lost elephant. The rest of the procedure followed the procedure of Experiment 1 except that the Ambiguous Pair contained the small elephant and a small pig and the Unambiguous Pair contained the large and small elephant. At the end, children were asked, “Which box do you want to open to help find the missing baby elephant?” See Figure 1, main text.

The *Similarity Judgment* task verified that children judged that elephants differing in size were more similar than a small elephant and small pig. The experimenter placed the small elephant and the small pig on the table next to each other and placed the large elephant and the small elephant next to each other approximately 18 cm away from the elephant/pig pair. The experimenter introduced the objects in pairs: “Here are two sets of objects. This set has this animal and this animal” (pointing to one set) “and this set has this animal and this animal” (pointing to the other; order and left/right position counterbalanced). The experimenter asked the child, "Which of these sets of things is more similar? Which set is more the same?"

Results

Children’s responses were coded online by the experimenter and recoded from video by a second coder blind to condition. Note that although the results were coded blind to condition (here and in the remaining studies), the experimenter was not herself blind to condition: she both demonstrated the items to the child and placed them in the box and thus knew which was the more discriminable contrast so we cannot absolutely rule out the possibility of experimenter influence. To mitigate this, the experimenter was trained to present the results neutrally throughout and looked directly at the child rather than at either box when asking the target question.

For the *Discrimination* task, children’s answers were coded as in Experiment 1; for the *Similarity Judgment* task, children responded by pointing at one of the sets or verbally indicating their choice (e.g. “the elephants”) and were coded as such.

In the *Discrimination* task, children behaved as in Experiment 1: nineteen out of twenty-four children successfully chose the box with the more discriminable pair (79.2%; 95% CI [0.63, 0.96]); the remaining five chose the box with the less discriminable pair. The *Similarity Judgment* task revealed that these results were not due to children thinking that the large and small elephant were most dissimilar overall: twenty of twenty-four children judged the large and small elephant to more similar to each other than the small elephant and small pig (83%; 95% CIs [0.67, 0.96]).

*Experiment 3*

Participants

Twenty-seven children were recruited; three participants were excluded from analysis, one due to experimenter error and two for failing the inclusion trial (see below), resulting in a sample of twenty-four children (mean age: 5;0; range 4;0-5;11). We restricted the age range to children four and up in this and the following experiments because accurate numerosity judgments were critical to the tasks and three-year-olds’ ability to count is fragile (e.g., *10*).

Materials

The materials used in the preliminary *Object Identity* experiment were used here for an inclusion task. In addition, in Experiment 3, four transparent cylinder tubes were used. Two tubes each contained eight different colored marbles, arranged in order to look identical to each other; one tube contained two white marbles, and one tube contained six white marbles. The tubes were sealed at the top with packing tape. Drawings of each of the marble tubes were used as a memory cue. The bunny puppet (henceforth referred to as Bunny to denote agency) used in Experiment 1 was also used here to occupy the children’s hands, limit interference, and as the “owner” of the smaller number in the pair of marbles in the experiment (see below).

Procedure

All children were tested individually in a private testing room off of the museum floor. The child and the experimenter sat on opposite sides of a child-sized table. All sessions were videotaped.

Children were introduced to the Bunny puppet “who will be playing some games with us.” Because we needed children to distinguish “their marbles” (the target set of marbles) from “Bunny’s marbles” (the distractor set), we used the ability to make this distinction as an inclusion criterion. The experimenter introduced the ball and the beanbag as in the preliminary *Object Identity* task. Children were asked which object they preferred. Whichever object the child chose, the Bunny announced that she preferred the other object. Each object was placed in a box behind the occluder (as in Experiment 1). After shaking each box, children were asked to choose the box that had “their object in it”. They were given a sticker for successfully choosing the box containing their choice. All but two children succeeded on this task; children who failed the task were excluded from analysis and replaced.

Next, the experimenter displayed the four tubes, prepared as described above. Bunny expressed a preference for the white marbles, touching the appropriate tubes and exclaiming, “White marbles! I love these white marbles!” The experimenter indicated the two tubes containing 8 colorful marbles and said, "See these marbles of different colors? For this game, these are yours! You're going to try to find *your* colorful marbles."

The experimenter described the hiding game. Children were told that one tube of marbles would be hidden inside each box. For the Ambiguous box, the possible contents were 6 white marbles or 8 colorful marbles; for the Unambiguous box, the possible contents were 2 white marbles or 8 colorful marbles. The experimenter placed the pictures depicting the possible contents of the two boxes on the table. The experimenter then introduced the occluder and mimed pouring the marbles out of the closed tube of eight marbles behind the occluder; no marbles exited the tube and each box was preloaded with eight marbles. After removing the screen, the experimenter reminded children about the possible contents of each box by pointing to the cartoon pictures: for the Unambiguous box, the experimenter said, “Remember, in *this* box there could be your marbles” (indicating the picture of the eight colorful marbles), and, “Or there could be Bunny's marbles” (indicating the picture of the two white marbles); for the Ambiguous box, the experimenter said, “And remember, in *this* box there could be your marbles” (indicating the picture of the colorful 8 marbles), “Or there could be Bunny's marbles” (indicate the picture of the 6 white marbles); left/right position and order counterbalanced throughout. The experimenter shook each box twice. She repeated the reminder about the possible box contents and shook the boxes again, twice. The experimenter asked children, “Which box do you want to open to find your colorful marbles?'' See Figure 1, main text.

Results

Children’s responses were coded live by the experimenter and recoded by a second coder blind to condition from video.

Eighteen out of twenty-four children successfully chose the box that could have contained the eight or two marbles – the more discriminable box – while six children chose the box that could have contained the eight or six marbles – the less discriminable box (75%; 95% CIs [0.58, 0.92])).

***Additional work***

In addition to Experiments 1-3, we ran an additional study to see if children could infer the discriminability of the hypotheses without hearing the sound of the marbles shaken in the box at all. We used a method identical to Experiment 3 except that the experimenter never hid the box, put the marbles in the box, or shook the boxes; instead children were simply asked from the outset which pair of marbles they wanted to use for the box-shaking discrimination game, either a difficult to discriminate pair consisting of 8 and 6 marbles or an easy to discriminate pair consisting of 8 and 2 marbles.

In the first iteration of this experiment, 13 out of 16 children chose the unambiguous pair, but this effect did not replicate in a pre-registered additional sample of 24 children (15 children chose the unambiguous pair). Without any perceptual experience of the sounds of the marbles, it may have been difficult for children to reliably simulate the possible outcomes and the relative difficulty of the discriminations, or the simulations may have been too coarse to guide their explicit choice of which task to select. Alternatively, it’s possible that after the simple warm-up task (Preliminary experiment, Object Identity), some children wanted a more challenging box-shaking game; they may have been sensitive to the difficulty of the discrimination, but, having not yet heard the sounds in the boxes, purposefully selected the harder game because it seemed more interesting.

**Experiments 4-7**

*Experiment 4*

Participants

Participants were recruited from an urban children’s museum. Consistent with the previous studies, we estimated the effect size (*f*) for a single experiment as 0.29. We used the power calculation program, G*Power, to calculate the planned sample size of for this experiment using *f* = 0.29, alpha = 0.05, and power = 0.80. The projected sample size using these values is 24 participants. Twenty-four children (mean age = 5;9; range 4;1-8;2) were included in the final sample. One additional child was excluded because they did not explore before providing a response on one or more trials (see Procedure for details).

Materials

A box covered with black electrical tape (18 cm x 16 cm x 12 cm) was used. Four objects were used in the practice trials: a plastic duck, a star-shaped pillow, a flat glass bead, and a cotton ball. For the test trials, standard-size glass marbles in eight colors and eight translucent cylindrical tubes were used. The tubes were pre-loaded with the appropriate number of marbles and sealed at the top; although children were told that the tubes of marbles would be poured into the box, marbles were in fact added quietly by hand to ensure that children did not get any evidence about the sound until they themselves shook the box.

A large cardboard screen (80 x 60 cm) was used both as an occluder and as an answer board with six Velcro tabs for children to provide their responses. Laminated pictures with Velcro tabs on the back, approximately to scale, were used to depict the possible contents of the box for both the practice trials and the test trials. A button was used to activate “hiding music” (the Jeopardy theme song) from a portable speaker, to mask any sound of marbles being placed into the hiding box.

In addition to measuring children's exploratory behavior via video coding, we developed an independent measure based on the time course of the motion of the box. We equipped a microcontroller with an accelerometer and placed the device in a small compartment of the box (the compartment was attached at a top corner of the box so as to minimize the possibility that it might interfere with box shaking). Custom Arduino software wirelessly transmitted the accelerometer readings, in real time, to a computer that recorded the measurements via a custom Python 2.7 program. The experimenter pressed a button at the start and end of every trial to record the time interval during which box shaking could have occurred.

Procedure

Children were introduced to the task as a guessing game in which their goal was to figure out what was hidden in the box. Two practice trials were used to teach children that 1) there were two possibilities for what could be hidden inside the box; 2) that these would be represented by the laminated pictures; 3) that children could not open the box but could shake the box or explore it in any other way they liked; 4) that they could make a guess by affixing one of the two pictures to the answer board, and 5) that they would not get feedback on every trial but would get feedback at the end of a set of trials (i.e., on the second of the two practice trials and on the last experimental trial).

The experimenter explained the practice task by introducing one set of practice objects (order counterbalanced). She said, “We’re going to play a guessing game. See these two toys? Do you want to feel them? I’m going to hide one of these toys inside the hiding box. Then you’re going to shake it and listen and see if you can figure out what’s inside. Remember, I’m going to hide either the (pillow or duck; bead or cotton ball) and you’re going to figure out what’s inside without opening the box!” Then the experimenter set up the answer board/occluding screen and placed the pictures of the two possible contents of the box on two Velcro tabs on the bottom of the screen facing the child. She pointed to each of the pictures in turn while reminding the child “I’m going hide either the (pillow or duck; bead or cotton ball) inside the box.” The experimenter then moved behind the occluding screen and placed one of the two objects into the box out of the child’s line of sight. To mask any acoustic cues generated by the experimenter (e.g. pouring the marbles into the box), the “hiding music” was played while the experimenter loaded the box with one set of marbles (counterbalanced across participants). The experimenter reminded the child of what could be inside of the box and indicated the location on the screen where the child could point the picture corresponding to his/her guess, and then handed the child the box. Children were allowed to shake or explore the box in any way they liked for as long as they liked until they made a verbal guess or touched a picture on the board.

Children did not receive any feedback on their guesses on the first practice trial. After the second practice trial, children were told that they were done with the first part of the game. The experimenter revealed the contents of the second box, and the children received a sticker for guessing correctly. (A few children guessed incorrectly on the second practice trial but were told they received the sticker for guessing correctly on the first box.)

Test trials were administered in the same manner as the practice trials, except that test trials consisted of contrasts of sets of marbles. The experimenter began each test trial by introducing two tubes of marbles. The contents of each tube differed from each other in color and each tube had a different number of marbles inside. See Figure 2, main text. The experimenter asked the child to count the number of marbles in each tube. The contrasts used for each experiment are displayed in Table 1. Trial order was counterbalanced, as was the order of introduction of the tubes of marbles, and the actual hidden contents of the box (e.g., whether 1 or 7 marbles were hidden inside on the 7 vs. 1 trial). As in the practice trials, children were allowed to shake or manipulate the box in any way they liked for as long as they liked until they made a guess about the contents of the box.

|  | Contrast 1 | | Contrast 2 | | Contrast 3 | | Contrast 4 | |
| --- | --- | --- | --- | --- | --- | --- | --- | --- |
| Experiment | Sets | d'^'^ | Sets | d'^'^ | Sets | d'^'^ | Sets | d'^'^ |
| Exp. 4 | 7 v 1 | 2.99 | 5 v 2 | 1.41 | 6 v 4 | 0.62 | 4 v 3 | 0.44 |
| Exp. 5 | 9 v 1 | 3.38 | 7 v 2 | 1.93 | 4 v 2 | 1.07 | 9 v 8 | 0.18 |
| Exp. 6 | 8 v 2 | 2.13 | 9 v 3 | 1.69 | 8 v 6 | 0.44 | 4 v 3 | 0.44 |
| Exp. 7 | 8 v 1 | 3.20 | 7 v 2 | 1.93 | 6 v 3 | 1.07 | 5 v 4 | 0.34 |

**Supplementary Table 1.** Contrasts used in Experiments 4-7, ordered from most discriminable to least discriminable based on the discriminability index (d') for each contrast derived from adult psychophysical data.

Results

Exploration time was coded from video by a human coder blind to contrast and, independently, by a motion sensor in the box (see SI). The behavioral coding included the time from the moment the child first contacted the box until she identified the contents of the box on each trial. The motion sensor coded the time from the initial motion to the final motion on each trial. We also looked at the motion sensor data including only time when the box was actually in motion (i.e., excluding any pauses; see SI). Here we report the results of the behavioral coding since the relationship between uncertainty and exploration may be best indexed by including time the children could have been planning subsequent actions and thinking about the data they generated but the primary results hold for all measures.

To normalize for individual differences in children’s exploratory behavior, we computed the time each child spent exploring on each trial as a proportion of the child’s total playtime across all four trials, and multiplied this proportion by the number of trials *k* in the experiment:

For trial *t*,

$transformed playtime of trial t=k * \frac{playtime of trial t}{sum of playtime across all k trials}$. (2)

In the current study, *k* = 4, but future work could explore experiments with different numbers of trials and multiplying the proportion by *k* provides a *k*-independent metric. Thus, a proportion less than 1 represents less playtime than would be expected if length of exploration was determined by chance, and a proportion greater than 1 represents more playtime that would be expected at chance. Although we transformed playtime to control for individual differences, the results of all model comparisons hold when using untransformed playtime reported in log seconds (the logarithmic transform was necessary to ensure normality). The children’s raw playtime was not normally distributed, violating the assumptions of our statistical tests so we only considered inferential statistics on log-transformed playtime (which is normally distributed).

As described in the main text, we estimated the difficulty of each contrast by constructing a model of children’s internal numerical representation and applying signal detection theory. We modeled the internal representation for each auditorily perceived number as a normal distribution on a log scale with equal variances $\sigma$ but logarithmically spaced means. Following (*2*), we constructed the probabilistic representations of auditorily perceived number depicted in Supplementary Figure S1; we show the mental representation in the original linear numerosity space for ease of visualization. We then computed the discriminability of each contrast between *l* and *m* marbles presented in Experiments 4-7 in terms of

$d^{'}=\frac{{|\mu}_{l}- \mu_{m}|}{\sigma}$, (3)

where $\mu_{l}=\log l$ and $\mu_{m}=\log m$ (*3*). Finally, we modeled children’s play time as a linear function of contrast difficulty, or negative discriminability, ${-d}^{'}$. For concreteness, we set$\sigma$ = 0.65, a coarse estimate based on both psychophysical studies of approximate number discrimination in children (*4; 5*) as well as the discrimination accuracies of children across Experiments 4-7. However, none of our model fits or quantitative predictions depend on this choice: Because our model of playtime is invariant to linear rescaling of $d^{'}$, its predictions are independent of the value of $\sigma$ and vary only with the difference in log numbers of marbles.

An alternative proposal for internal representation of number assumes normal distributions over *linear* space, with the variance of each normal distribution proportional to its mean (*6*); see Supplementary Figure S1B. As we show below this metric produces nearly identical results to the one described above, but we prefer the logarithmic representation to the linear representation because the latter truncates the representation at zero and therefore does not allocate equal probability to each normal distribution. Still, we can compute *d’* in the linear representation using the conventional estimator for unequal variances,

$d^{'}=\frac{\left| \mu_{l}- \mu_{m} \right|}{\sqrt{\frac{1}{2}\left( \sigma_{l}^{2}+\sigma_{m}^{2} \right)}}= \frac{1}{w}\times\frac{\left| l-m \right|}{\sqrt{\frac{1}{2}\left( l^{2}+m^{2} \right)}}$ , (4)

where *w* is a constant that determines how variance grows with mean and *l* and *m* denote different numbers of marbles. We set *w* = 0.4, again based on both previous psychophysical studies of approximate number discrimination (*4, 5*) and our discrimination accuracies, but as in the logarithmic representation above our predictions for children’s playtimes do not depend on *w* because they are invariant to linear rescaling of *d’.* See Supplementary Figure S2B for evaluation of this metric.

Finally, we also considered an alternative difficulty metric, *b’*, that is inspired by *d’* (and uses the same functional form) but can be defined behaviorally from numerical estimation judgments rather than from a model of internal sensitivity. We computed the difficulty of each contrast from judgments that adult participants made in a related task: estimating the exact number of marbles in a box that was shaken, from pre-recorded sounds of marbles shaken by the experimenter for a fixed 2-second interval (*7*). We calculated the mean and standard deviation of participant responses for each of 1-9 marbles, and calculated *b’* (using the same function as unequal-variance *d’* above):

$b^{'}=\frac{\left| \mu_{l}- \mu_{m} \right|}{\sqrt{\frac{1}{2}\left( \sigma_{l}^{2}+\sigma_{m}^{2} \right)}}$ (5)

for each *l*, *m* numerosity contrast.

**Supplementary Figure 1.** Visualization of models of children’s internal representation of number, showing (A) normal distributions with fixed variance defined over logarithmic space (but visualized over linear space) and (B) normal distributions with variance proportional to mean defined over linear space.

Using the R (version 3.6.3) programming language (*46*), the data were submitted to linear mixed-effects regression models, with subject as a random effect. An example of our model specification (with discriminability as a predictor variable) in the common lme4 (*47*) syntax is as follows: Playtime ~ Discriminability + (1 | subject). We ran four models with the following predictors: 1) Discriminability; 2) Trial order; 3) Discriminability + Trial order; 4) Discriminability + Trial order + Number of marbles inside the box. To assess which of these variables predicted significant variance, we ran three model comparisons using the R anova function. This allowed us to obtain p-values from likelihood ratio tests of the full model with the effect in question against the model without the effect in question^[[1]](#footnote-1)^. Comparing Models 1 and 3, we found that trial order had a significant effect on exploration time, where children on average explored for less time as the task progressed, χ2(1)=5.95, *p* < 0.05 (and a marginal effect when considering the untransformed log playtime measure: χ2(1)=3.70, *p=*0.055). Comparing Models 2 and 3, we found that discriminability affected children’s exploration time, where the less discriminable the contrast, the more children explored, χ2(1)=16.23, *p* < 0.0001 (untransformed log playtime: χ2(1)=15.07, *p* < 0.005). This model comparison shows that discriminability explains variance above and beyond the effect of trial order. Comparing Models 3 and 4, we found no effect of the number of marbles inside the box, suggesting children’s exploration time was not affected by what they actually heard, but rather by the discriminability of the two sets, χ2(1)=0.26, *p=*0.48 (untransformed log playtime: χ2(1)=0.72, *p=*0.40). In addition, we bootstrapped 95% confidence intervals of mean exploration time to assess overlap across the four contrasts. We found that the most discriminable contrast’s confidence interval did not overlap with the intervals of the two least discriminable contrasts. The second most discriminable contrast overlapped with the other three contrasts (See Fig. 2). The same pattern of results held when considering untransformed log playtime. These results provide preliminary evidence that children’s exploration is well-calibrated to the discriminability of the hypotheses under consideration.

*Experiment 5*

Experiment 5 was identical to Experiment 4 except for the set of contrasts used, see Table 1. Twenty-four children (mean = 5;11; range 4;1-8;0) were recruited and participated.

Results

Data were coded as in Experiment 4. Again, to normalize for individual differences in children’s exploratory behavior, we computed the time each child spent exploring on each trial as a proportion of the child’s total playtime across all four trials. The same models were used as in Experiment 4. Like in Experiment 4, we that trial order had a significant effect on exploration time, χ2(1)=0.11, *p=*0.74 (untransformed log playtime: χ2(1)=0.10, *p=*0.75). Our key prediction, that discriminability predicts children’s exploration time replicated in Experiment 5, χ2(1)=19.53, *p* < 0.0001 (untransformed log playtime: χ2(1)=15.49, *p* < 0.0001). Once again, we found no effect of the number of marbles inside the box, χ2(1)=0.22, *p=*0.64 (untransformed log playtime: χ2(1)=0.0055, *p=*0.94). Comparing bootstrapped 95% confidence intervals of mean playtime, we found that the most discriminable contrast’s confidence interval did not overlap with the intervals of the two least discriminable contrasts. The second most discriminable contrast overlapped with the other three contrasts (See Fig. 2). The same pattern held for untransformed log playtime. These results again suggest that children’s exploration is closely matched to the difficulty of the discrimination problem.

*Experiment 6*

The same procedure as in the preceding experiments was used except for the contrasts (from most to least discriminable 8 vs. 2; 3 vs. 9; 8 vs. 6; and 3 vs. 4); also, rather than counterbalancing the number of marbles in the box, there were always either 8 or 3 marbles hidden in the box. This provides a strong test of whether children’s exploration is driven primarily by the salience or ancillary sensory properties of the stimuli. If so, children should spend more time exploring the box when it contained more (or fewer) marbles. If instead, children’s exploration tracks not the actual contents of the box but the discriminability of the actual contents from the alternatives, then children should spend proportionately less time exploring on the two easy contrasts (8 vs. 2 and 3 vs. 9) than the two hard ones (8 vs. 6 and 3 vs. 4). Twenty-four children (mean = 5;9, range 4;1-7;8) were included in the final sample. Three additional children were excluded because of family interference (*n* = 1) and issues with video recordings (*n* = 2).

Results

Data were coded as in previous experiments. Again, to normalize for individual differences in children’s exploratory behavior, we computed the time each child spent exploring on each trial as a proportion of the child’s total playtime across all four trials. The same models were used. As in Experiment 4, we found that trial order also had a significant effect on exploration time, χ2(1)=14.03, *p* < 0.0005 (untransformed log playtime: χ2(1)=11.77, *p* < 0.01). As in Experiments 4 and 5, we found that discriminability was a significant predictor of children’s exploration time, χ2(1)=12.35, *p* < 0.0005 (untransformed log playtime: χ2(1)=8.10, *p* < 0.005). Experiment 6 provided a strong test of whether the number of marbles heard inside the box affects exploration time since two hard discrimination trials (8 vs. 6 and 3 vs. 4) and two easy discrimination contrasts (8 vs. 2 and 3 vs. 9), were matched for the number of marbles inside the box. We found no effect of the number of marbles inside the box, χ2(1)=1.19, *p=*0.28 (untransformed log playtime: χ2(1)=0.96, *p=*0.33). In addition, we bootstrapped 95% confidence intervals of mean exploration time to assess overlap across the four contrasts. We found that the most discriminable contrast’s confidence interval did not overlap with the intervals of the two least discriminable contrasts. The second most discriminable contrast overlapped with the other three contrasts (see Fig. 2). The same pattern of results held for the untransformed log playtime metric.

*Experiment 7*

To establish the robustness of the pattern of results in Experiments 4-6, we pre-registered all methods and analyses on the Open Science Framework for Experiment 7 and the joint analysis to follow. The same procedure as in the preceding experiments was used (see Supplementary Table S1 for contrasts). Participants were recruited from an urban children’s museum. Twenty-four children (mean = 5;11; range 4;3-7;8) were included in the final sample. One additional child was excluded due to attention issues.

Results

Data were coded and normalized as in previous experiments, and the same models were used. Unlike in Experiments 4 and 6, but as in Experiment 5, trial order had no effect on exploration time, χ2(1)=0.011, *p=*0.92 (untransformed log playtime: χ2(1)=0.0010, *p=*0.98). As in Experiments 4-6, discriminability was a significant predictor of children’s exploration time, χ2(1)=14.75, *p* < 0.0005 (untransformed log playtime: χ2(1)=13.76, *p* < 0.005) and there was no effect of the number of marbles inside the box, χ2(1)=0.21, *p=*0.64 (untransformed log playtime: χ2(1)=0.52, *p=*0.47). In addition, we bootstrapped 95% confidence intervals of mean exploration time to assess overlap across the four contrasts. We found that the most discriminable contrast’s confidence interval did not overlap with the interval of the least discriminable contrast. The second most discriminable contrast overlapped with the other three contrasts (see Fig. 2). As in Experiment 6, the confidence intervals of all four contrasts overlapped when considering untransformed log playtimes.

*Joint analysis*

Our primary analysis, as reported in the main text of the manuscript and pre-registered on the Open Science Framework, looked at the quantitative relationship between discriminability and children’s exploration time over all 16 contrasts in Experiments 4-7. This analysis used the same linear mixed-effects models that we evaluated for the individual experiments, with an additional random effect for Experiment. Looking at the same three model comparisons that we analyzed for individual experiments, we found an effect of trial order, χ2(1)=8.63, *p* < .005 (untransformed log playtime: χ2(1)=6.76, *p* < 0.01) and discriminability, χ2(1)=63.92, *p* < 0.00001 (untransformed log playtime: χ2(1)=56.97, *p* < .00001), but no effect of marbles in the box, χ2(1)=0.124, *p*=0.72 ( untransformed log playtime: χ2(1)=0.87. Supplementary Table S2 displays the regression table for the best performing model (Model 3, with fixed effects for Discriminability and Trial number and a random effect for Experiment).

Also, as noted in the main text, in addition to analyzing the behavioral data, we conducted the same joint analysis for the motion sensor data^[[2]](#footnote-2)^; we did this both including all motion from the first to last movement of the box and excluding times when the box was still (i.e., including only times when the box was actually in motion). These two coding methods yielded comparable estimates for the effect of discriminability on exploration time (including times when the box was still: β=0.10, 95% CI [0.05, 0.13]; excluding same: β**=**0.086, 95% CI [0.051, 0.12]). Children’s exploration times also correlated similarly with the discriminability of the contrast under both coding methods (including: *r=*0.89; 95% CI [0.55, 0.89]; excluding: *r*=0.86; 95% CI [0.54, 0.88]). See Supplementary Fig. S1. For ease of comparison, we duplicate Figs. 3A and 3B from the manuscript as Supplementary Fig. S1A and S1B here; Supplementary Fig. S1C shows results including only times when the box was in motion.

*Additional Heuristic Models*

We examined two potential heuristics that might underlie children’s exploratory behavior. First, we considered whether a very simple cue, the difference between the number of marbles in each hypothesis (tube), could explain children’s behavior. Formally we define the numerical difference heuristic as

$nd= \left| l-m \right|$, (6)

where *l* and *m* are the number of marbles in a given contrast. $nd$ is intuitively related to discriminability; a larger value indicates high discriminability, and a smaller value low discriminability (the exact relationship is unclear but we expect $nd$ to increase monotonically with discriminability).

Second, we examined another alternative heuristic that takes the ratio of the larger to the smaller number of marbles as a predictor of exploration time. This heuristic formalizes the intuition of “distance from 50-50 split” – how far away a given pair is from having the same number of marbles in each set. Formally we define the numerical ratio heuristic as the ratio

$nr= \frac{-l}{m}$, (7)

where *l* is the smaller and *m* is the larger number of marbles in a given contrast.

Both $nd$ and $nr$ are good quantitative predictors of children’s box shaking time ($nd$: *r=*0.94, 95% CI [0.76, 0.94], $nr$: *r*=0.95, 95% CI [0.78, 0.95]). The fit of the $nr$ heuristic is numerically indistinguishable from the *d’* measure we use; this should not be surprising as there is a close correspondence between the mathematical structure of these two measures, and they are themselves correlated at *r*=0.96. The $nd$ heuristic performs slightly worse, but there is a qualitative difference between its predictions and those of *d’* or $nr$. Across Experiments 4-7, there are four subsets of stimuli where the numerical difference is constant but discriminability *d’* and the numerical ratio *nr* differ, and intuitively the task seems more difficult when *d’* or $nr$ are smaller: e.g., a numerical difference of 2 occurs with both contrasts of 4 v 2 marbles and 8 v 6 marbles, but 8 v 6 seems much more difficult than 4 v 2. This intuition is borne out by our empirical results. For contrasts scored equally by $nd$ but not by *d’*, children on average explored more when the contrasts were less discriminable. Indeed for each of the four numerical differences shared by more than one contrast, regression analysis revealed a positive relationship between exploration time and negative discriminability (Supplementary Figure S4). Because each numerical difference corresponded only to at most four contrasts, none of these linear relationships is statistically significant on its own, but the overall pattern of a positive relationship in all four out of four possible subsets of contrasts is strongly suggestive of an effect of discriminability independent of absolute numerical difference.

Unlike $nd$, $nr$ makes different predictions for different contrasts with the same numerical difference, in ways that are almost perfectly correlated with of *d’*. We therefore suggest that if a numerical heuristic turns out to provide the best explanation of children’s box-shaking behavior – that is, if children were in fact explicitly estimating discriminability from the numbers of marbles shown rather than judging the discriminability of imagined perceptual evidence from alternative hypotheses via mental simulation – $nr$ would be a more plausible heuristic account than $nd$. Because $nr$ is so closely related to *d’* it might even serve as a resource-rational approximation of the ideal *d’.*

­­­


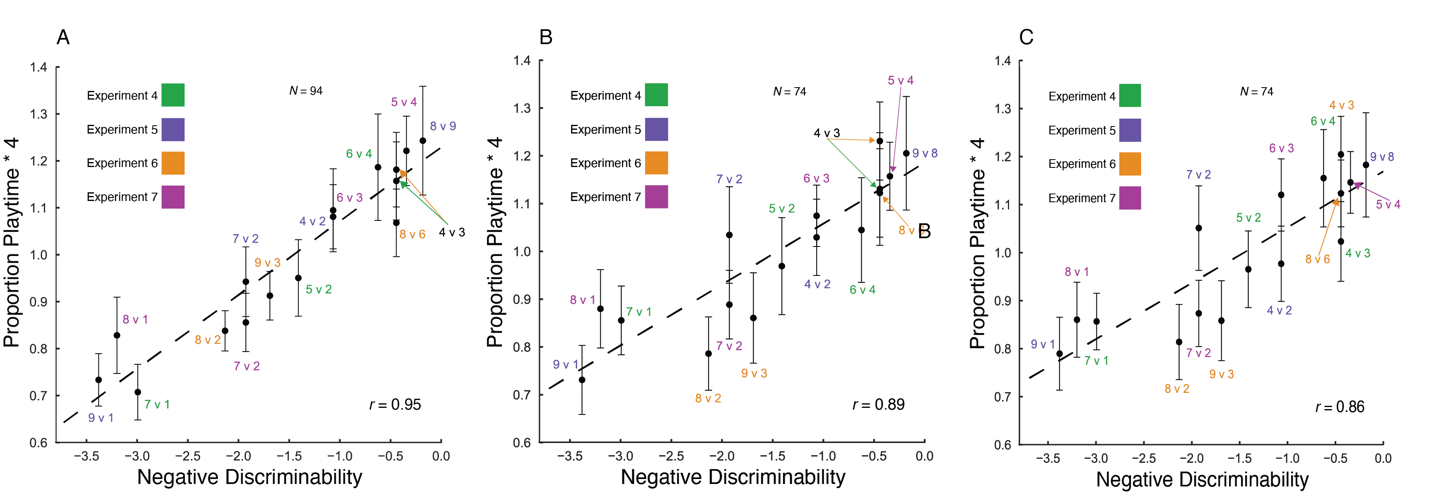


**Supplementary Figure 2**. Children’s proportional exploration times as a function of the negative discriminability of each contrast across Experiments 4-7, showing data coded (a) from video, and from motion sensor (b) including and (c) excluding times when the box was not in motion. Source data are provided as a Source Data file.


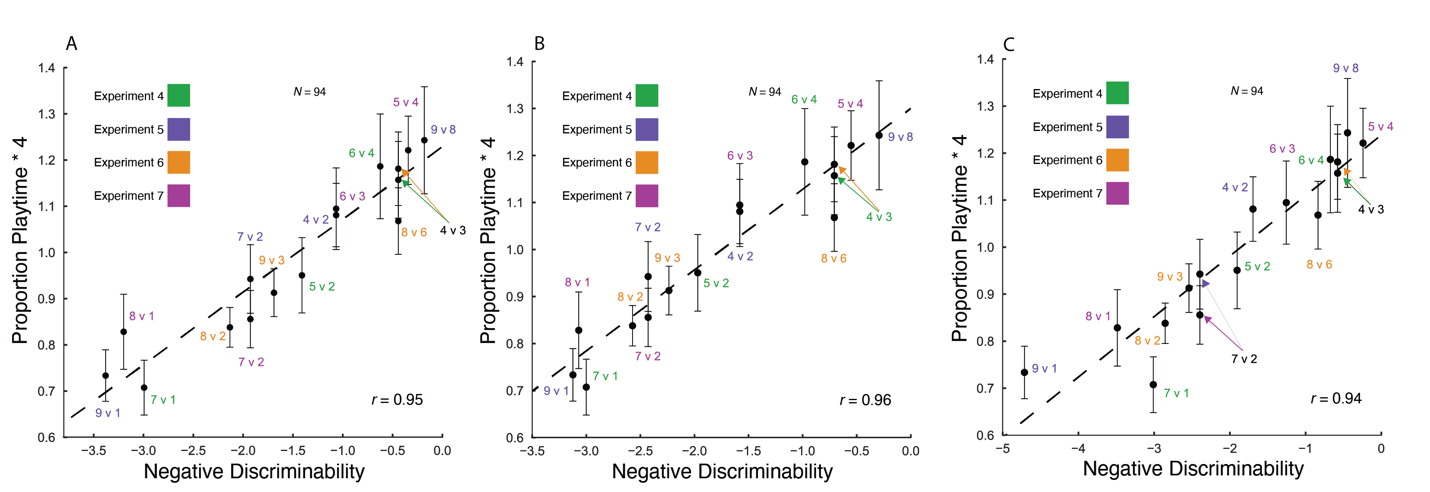


**Supplementary Figure 3.** Results of alternative modeling approach, showing *d’* calculated using (a) the logarithmic representation adopted in the main text, (b) an alternative representation with linearly increasing means and variances (with numerosity), and (c) a related measure, *b’*, estimated from adult subjects. Source data are provided as a Source Data file.

|  | Estimate | Standard error | Degrees of freedom | *t* | *p* < |
| --- | --- | --- | --- | --- | --- |
| Discriminability | 0.15 | 0.19 | 381.00 | 8.31 | 1 × 10^-15^ |
| Trial | -0.05 | 0.17 | 381.00 | -2.94 | 0.005 |

**Supplementary Table 2**. Regression table for the best performing linear model, Model 3. Source data are provided as a Source Data file.

**
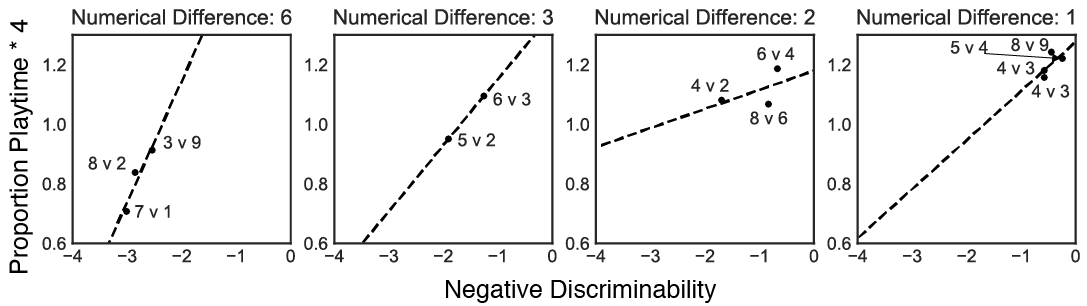
Supplementary Figure 4.** Children's exploration time as a function of negative discriminability d', for a given numerical difference $nd$ between elements of a contrast.  Subplots show four subsets of stimuli across Exps 4-7 where d' varies for a given value of $nd$ for four different values of $nd$.  In all four cases, exploration time tends to increase with d' even though numerical difference is fixed, suggesting that children are sensitive to the psychophysical discriminability of contrasts beyond what is captured by the simple numerical difference measure. Source data are provided as a Source Data file.

**Supplementary Information References**

1. Hagedorn, J., Hailpern, J., Karahalios, K.G. (2008) VCode and VData: Illustrating a new Framework for Supporting the Video Annotation Workflow, in *Proceedings of the working conference on Advanced Visual Interfaces* (ACM, Napoli), 317-321.

2. Dehaene, S., Mehler, J. (1992). Cross-linguistic regularities in the frequency of number words. *Cognition*, *43*(1), 1-29.

3. Dehaene, S. (2007). Symbols and quantities in parietal cortex: Elements of a mathematical theory of number representation and manipulation. *Sensorimotor foundations of higher cognition*, *22*, 527-574.

4. Halberda, J., Odic, D. (2015). The precision and internal confidence of our approximate number thoughts. In *Mathematical Cognition and Learning* (Vol. 1, pp. 305-333). Elsevier.

5. Halberda, J., Feigenson, L. (2008). Developmental change in the acuity of the" Number Sense": The Approximate Number System in 3-, 4-, 5-, and 6-year-olds and adults. *Developmental psychology*, *44*(5), 1457.

6. Gallistel, C. R., Gelman, R. (1992). Preverbal and verbal counting and computation. *Cognition*, *44*(1-2), 43-74.

7. Siegel, M.H., Tenenbaum, J.B., McDermott, J.H. (2018) Physical inference for object perception in complex auditory scenes. Paper presented at the 40^th^ Annual Conference of the Cognitive Science Society.

8. Ihaka, R., Gentleman, R. (1996) R: A Language for Data Analysis and Graphics. *Journal of computational and graphical statistics*, **5**:299-314.

9. Bates, D., Mächler, M., Bolker, B., Walker, S. (2015) Fitting Linear Mixed-Effects Models Using lme4. *Journal of statistical software*, **67**:1-48.

10. Sarnecka, B. W., & Carey, S. (2008). How counting represents number: What children must learn and when they learn it. *Cognition*, **108**:662-674.

1. A detailed description of the analyses is available on the Open Science Framework at the following current link: <https://osf.io/vnzbr/?view_only=ba3ca1c5ff9346c0a39e731291aa5d5f> [↑](#footnote-ref-1)
2. Because of technical difficulties, 22 of the 96 trials lacked motion data and were not included in the analysis of the motion sensor data. [↑](#footnote-ref-2)
