# Supplementary material for: Children’s exploratory play tracks the discriminability of hypotheses
Source: Nat Commun. 2021 Jun 14;12:3598. doi: 10.1038/s41467-021-23431-2 (PMC8203670; doi:10.1038/s41467-021-23431-2)
Supplement: Supplementary file 3 — Reporting Summary [file 41467_2021_23431_MOESM3_ESM.pdf]

nature research

Corresponding author(s): NCOMMS-19-26073B

Last updated by author(s): 2/26/2021

Reporting Summary

Nature Research wishes to improve the reproducibility of the work that we publish. This form provides structure for consistency and transparency in reporting. For further information on Nature Research policies, see our Editorial Policies and the Editorial Policy Checklist.

Please do not complete any field with "not applicable" or n/a. Refer to the help text for what text to use if an item is not relevant to your study. For final submission: please carefully check your responses for accuracy; you will not be able to make changes later.

Statistics

For all statistical analyses, confirm that the following items are present in the figure legend, table legend, main text, or Methods section.

n/a	Confirmed	
☐	☒	The exact sample size (n) for each experimental group/condition, given as a discrete number and unit of measurement
☒	☐	A statement on whether measurements were taken from distinct samples or whether the same sample was measured repeatedly
☐	☒	The statistical test(s) used AND whether they are one- or two-sided<br>*Only common tests should be described solely by name; describe more complex techniques in the Methods section.*
☒	☐	A description of all covariates tested
☐	☒	A description of any assumptions or corrections, such as tests of normality and adjustment for multiple comparisons
☐	☒	A full description of the statistical parameters including central tendency (e.g. means) or other basic estimates (e.g. regression coefficient) AND variation (e.g. standard deviation) or associated estimates of uncertainty (e.g. confidence intervals)
☐	☒	For null hypothesis testing, the test statistic (e.g. F, t, r) with confidence intervals, effect sizes, degrees of freedom and P value noted<br>*Give P values as exact values whenever suitable.*
☐	☒	For Bayesian analysis, information on the choice of priors and Markov chain Monte Carlo settings
☒	☐	For hierarchical and complex designs, identification of the appropriate level for tests and full reporting of outcomes
☐	☒	Estimates of effect sizes (e.g. Cohen's d, Pearson's r), indicating how they were calculated

Our web collection on statistics for biologists contains articles on many of the points above.

Software and code

Policy information about availability of computer code

Data collection	All data, code, and analysis are available on the Open Science Framework:<br>https://osf.io/ytvse, https://osf.io/dxguw/,<br>https://osf.io/n97fr/
Data analysis	All data, code, and analysis are available on the Open Science Framework:<br>https://osf.io/ytvse/, https://osf.io/dxguw/<br>https://osf.io/n97fr/

For manuscripts utilizing custom algorithms or software that are central to the research but not yet described in published literature, software must be made available to editors and reviewers. We strongly encourage code deposition in a community repository (e.g. GitHub). See the Nature Research guidelines for submitting code & software for further information.

Data

Policy information about availability of data

All manuscripts must include a data availability statement. This statement should provide the following information, where applicable:

- Accession codes, unique identifiers, or web links for publicly available datasets
- A list of figures that have associated raw data
- A description of any restrictions on data availability

All material specifications and anonymized data (including data associated with Figures 3, 4, and 5) are available on the Open Science Framework (https://osf.io/ytvse/, https://osf.io/dxguw/, https://osf.io/n97fr/).

Field-specific reporting

Please select the one below that is the best fit for your research. If you are not sure, read the appropriate sections before making your selection.

☐ Life sciences ☒ Behavioural & social sciences ☐ Ecological, evolutionary & environmental sciences

For a reference copy of the document with all sections, see nature.com/documents/nr-reporting-summary-flat.pdf

Behavioural & social sciences study design

All studies must disclose on these points even when the disclosure is negative.

Study description

We looked at children's ability to distinguish competing hypotheses about the contents of a box by comparing the sounds they heard to a representation of the sounds they would have heard under alternative hypotheses. The data were both qualitative and quantitative.

Research sample

The sample consisted of children ranging from 2;7 to 8;3 across studies. The age was chosen to test both the earliest emergence and range of these abilities, which have not been previously assessed in children. The age range was informed by earlier work on children's understanding of numerosity and their metacognitive abilities. The same population (drawn from an urban children's museum) was sampled for all studies reported in this manuscript. Children were recruited from an urban children's museum. While most of the children were white and middle class, a range of ethnicities and socioeconomic backgrounds reflecting the diversity of the local population (47% European American, 24% African American, 9% Asian, 17% Latino, 4% two or more races) and the museum population (29% of museum attendees receive free or discounted admission) were represented. All parents provided written informed consent consistent with the MIT IRB approval for the study. Children over age seven also provided verbal assent.

Sampling strategy

All families with children in the target age range were approached on the museum floor. Within experiments, children were randomly assigned to condition. Based on the results of the preliminary experiments, we estimated the effect size for a single experiment as f = 0.29. We used the power calculation program G*Power to calculate the planned sample size of for this experiment using f = 0.29, α = 0.05, and power = 0.80. The projected sample size using these values was 24 participants,

Data collection

All children were tested individually in a private testing room off of the museum floor. The child and the experimenter sat on opposite sides of a child-sized table. The stimuli were boxes, marbles, a stuffed animal puppet, a cardboard screen and velcro pictures. All sessions were videotaped. Children's responses were coded live by the experimenter and recoded by a coder blind to condition from video. In addition to measuring children's exploratory behavior via video coding, we developed an independent measure based on the time course of the motion of the box. We equipped a microcontroller with an accelerometer, and placed the device in a small compartment of the box (the compartment was attached at a top corner of the box so as to minimize the possibility that it might interfere with box shaking). Custom software wirelessly transmitted the accelerometer readings, in real time, to a computer that recorded the measurements.

Timing

Data collection occurred from Summer 2015- Fall, 2018. Data collection was interrupted for some months in 2017 due to illness in the first author

Data exclusions

Exclusion criteria were pre-registered on OSF as follows:
a.Experimenter Error: If the experimenter incorrectly administers the task (e.g., explains the games incorrectly, asks the test questions incorrectly).
b.Family interference: If parent or sibling interferes in the task (e.g., telling the participant what to choose or distracting the child from the task by talking to them or touching the stimuli while the participant is playing the games responding to any of the verbal questions before the participant has a chance to respond).
c.Language issues: If the participant does not understand the task instructions because of language difficulties (e.g., not fluent in English).
d.Voluntarily withdrawing: If the participant or their parent/legal guardian chooses to withdraw before the task is complete or does not answer the test questions.
e.Attention Issues: If the child is not visibly attending to the Experimenter (e.g., hiding during the task, failing to interact with the stimuli by talking over the experimenter, choosing a game before the experimenter has asked the test question).
f.Failure to find their 'favorite' ball in the warm-up trial

Children were excluded from six of the seven experiments as follows:
Experiment 1 "Although we included two-year-olds in the preliminary experiments, we did not include them in the following studies because pilot work established that the task demands (requiring them to represent that one of two items could be placed in each box) were too high."
Experiment 2: "Fifty-two children were recruited; four participants were excluded from analysis, three because of experimenter error and one for inability to understand and follow directions."
Experiment 3: "Twenty-seven children were recruited; three participants were excluded from analysis, one due to experimenter error and two for failing the inclusion trial."
Experiment 4: "Twenty-four children (mean age = 5;9; range 4;1-8;2) were included in the final sample. One additional child was excluded because they did not explore before providing a response on one or more trials"

Experiment 5: No children were excluded
Experiment 6 :" Three additional children were excluded because of family interference (n = 1) and issues with video recordings (n = 2)."
Experiment 7: "Twenty-four children (mean = 5;11; range 4;3-7;8) were included in the final sample. One additional child was excluded due to attention issues."

Non-participation	No participants dropped out of the study or declined participation
Randomization	Participants were randomly assigned to experimental groups

Reporting for specific materials, systems and methods

We require information from authors about some types of materials, experimental systems and methods used in many studies. Here, indicate whether each material, system or method listed is relevant to your study. If you are not sure if a list item applies to your research, read the appropriate section before selecting a response.

Materials & experimental systems

n/a	Involved in the study	
☒	☐	Antibodies
☒	☐	Eukaryotic cell lines
☒	☐	Palaeontology and archaeology
☒	☐	Animals and other organisms
☐	☒	Human research participants
☒	☐	Clinical data
☒	☐	Dual use research of concern

Methods

n/a	Involved in the study	
☒	☐	ChIP-seq
☒	☐	Flow cytometry
☐	☐	MRI-based neuroimaging

Human research participants

Policy information about studies involving human research participants

Population characteristics	See above
Recruitment	As above, children were recruited from an urban children's museum. Researchers are trained to approach all families with children in the relevant age range were recruited from the museum floor without pre-judging the relationship of the adult to the children or the families' likelihood of participation.
Ethics oversight	MIT

Note that full information on the approval of the study protocol must also be provided in the manuscript.

Magnetic resonance imaging

Experimental design

Design type	*Indicate task or resting state; event-related or block design.*
Design specifications	*Specify the number of blocks, trials or experimental units per session and/or subject, and specify the length of each trial or block (if trials are blocked) and interval between trials.*
Behavioral performance measures	*State number and/or type of variables recorded (e.g. correct button press, response time) and what statistics were used to establish that the subjects were performing the task as expected (e.g. mean, range, and/or standard deviation across subjects).*

Acquisition

Imaging type(s)	*Specify: functional, structural, diffusion, perfusion.*
Field strength	*Specify in Tesla*
Sequence & imaging parameters	*Specify the pulse sequence type (gradient echo, spin echo, etc.), imaging type (EPI, spiral, etc.), field of view, matrix size, slice thickness, orientation and TE/TR/flip angle.*
Area of acquisition	*State whether a whole brain scan was used OR define the area of acquisition, describing how the region was determined.*

Diffusion MRI ☐ Used ☐ Not used

Preprocessing

Preprocessing software	*Provide detail on software version and revision number and on specific parameters (model/functions, brain extraction, segmentation, smoothing kernel size, etc.).*
Normalization	*If data were normalized/standardized, describe the approach(es): specify linear or non-linear and define image types used for transformation OR indicate that data were not normalized and explain rationale for lack of normalization.*
Normalization template	*Describe the template used for normalization/transformation, specifying subject space or group standardized space (e.g. original Talairach, MNI305, ICBM152) OR indicate that the data were not normalized.*
Noise and artifact removal	*Describe your procedure(s) for artifact and structured noise removal, specifying motion parameters, tissue signals and physiological signals (heart rate, respiration).*
Volume censoring	*Define your software and/or method and criteria for volume censoring, and state the extent of such censoring.*

Statistical modeling & inference

Model type and settings	*Specify type (mass univariate, multivariate, RSA, predictive, etc.) and describe essential details of the model at the first and second levels (e.g. fixed, random or mixed effects; drift or auto-correlation).*
Effect(s) tested	*Define precise effect in terms of the task or stimulus conditions instead of psychological concepts and indicate whether ANOVA or factorial designs were used.*

Specify type of analysis: ☐ Whole brain ☐ ROI-based ☐ Both

Statistic type for inference<br>(See Eklund et al. 2016)	*Specify voxel-wise or cluster-wise and report all relevant parameters for cluster-wise methods.*
Correction	*Describe the type of correction and how it is obtained for multiple comparisons (e.g. FWE, FDR, permutation or Monte Carlo).*

Models & analysis

n/a	Involved in the study
☐	☐ Functional and/or effective connectivity
☐	☐ Graph analysis
☐	☐ Multivariate modeling or predictive analysis

Functional and/or effective connectivity	*Report the measures of dependence used and the model details (e.g. Pearson correlation, partial correlation, mutual information).*
Graph analysis	*Report the dependent variable and connectivity measure, specifying weighted graph or binarized graph, subject- or group-level, and the global and/or node summaries used (e.g. clustering coefficient, efficiency, etc.).*
Multivariate modeling and predictive analysis	*Specify independent variables, features extraction and dimension reduction, model, training and evaluation metrics.*

his checklist template is licensed under a Creative Commons Attribution 4.0 International License, which permits use, sharing, adaptation, distribution and reproduction in any medium or format, as long as you give ppropriate credit to the original author(s) and the source, provide a link to the Creative Commons license, and indicate if changes were made. The images or other third party material in this article are included in e article's Creative Commons license, unless indicated otherwise in a credit line to the material. If material is not included in the article's Creative Commons license and your intended use is not permitted by atutory regulation or exceeds the permitted use, you will need to obtain permission directly from the copyright holder. To view a copy of this license, visit http://creativecommons.org/licenses/by/4.0/
